# *Pinelliae Rhizoma*, a Toxic Chinese Herb, Can Significantly Inhibit CYP3A Activity in Rats

**DOI:** 10.3390/molecules20010792

**Published:** 2015-01-07

**Authors:** Jinjun Wu, Zaixing Cheng, Shugui He, Jian Shi, Shuqiang Liu, Guiyu Zhang, Lijun Zhu, Liang Liu, Zhongqiu Liu, Na Lin, Linlin Lu

**Affiliations:** 1 International Institute for Translational Chinese Medicine, Guangzhou University of Chinese Medicine, Guangzhou 510006, China; E-Mails: wujinjun1018@163.com (J.W.); heshugui666@163.com (S.H.); shijian0225@126.com (J.S.); lshq120@163.com (S.L.); zhangguiyu0226@126.com (G.Z.); zhulijun115623@163.com (L.Z.); liuzq@gzucm.edu.cn (Z.L.); linna888@163.com (N.L.); 2 College of Pharmacy, Fujian University of Traditional Chinese Medicine, Fuzhou 350108, China; E-Mail: chengzaixing2086@sohu.com; 3 State Key Laboratory of Quality Research in Chinese Medicine, Macau University of Science and Technology, Macau, China; E-Mail: lliu@must.edu.mo; 4 Institute of Chinese Meteria Medica, China Academy of Chinese Medical Sciences, Beijing 100700, China

**Keywords:** *Pinelliae Rhizoma*, CYP3A, testosterone, buspirone, drug–drug interactions

## Abstract

Raw *Pinelliae Rhizoma* (RPR) is a representative toxic herb that is widely used for eliminating phlegm or treating cough and vomiting. Given its irritant toxicity, its processed products, including *Pinelliae Rhizoma Praeparatum* (PRP) and *Pinelliae Rhizoma Praeparatum cum Zingibere et Alumine* (PRPZA), are more commonly applied and administered concomitantly with other chemical drugs, such as cough medications. This study aimed to investigate the effects of RPR, PRP, and PRPZA on CYP3A activity. Testosterone (Tes) and buspirone (BP) were used as specific probe substrates *ex vivo* and *in vivo*, respectively. CYP3A activity was determined by the metabolite formation ratios from the substrates. *Ex vivo* results show that the metabolite formation ratios from Tes significantly decreased, indicating that RPR, PRP, and PRPZA could inhibit CYP3A activity in rats. CYP3A protein and mRNA levels were determined to explore the underlying mechanism. These levels showed marked and consistent down-regulation with CYP3A activity. A significant decrease in metabolite formation ratios from BP was also found in PRPZA group *in vivo*, implying that PRPZA could inhibit CYP3A activity. Conclusively, co-administration of PR with other CYP3A-metabolizing drugs may cause drug–drug interactions. Clinical use of PR-related formulae should be monitored carefully to avoid adverse interactions.

## 1. Introduction

*Pinelliae Rhizoma* (PR), the tuber of *Pinellia ternate* (Thunb.) Breit, is a commonly used Chinese herb with high bioactivity against cough and vomiting, or for eliminating the stagnation of phlegm [1,2,3]. PR is distributed in China, Korea, and Japan. It has been used as an essential drug in Chinese clinics for thousands of years. However, PR is also a representative toxic herbal medicine, which can cause irritant toxicity to oral, throat, and gastrointestinal mucosa [4,5,6]. Thus, PR is commonly used in its preparation forms in Chinese clinics. As recorded in the Chinese Pharmacopoeia (2005 and 2010 Edition), PR mainly comprises raw *Pinelliae Rhizoma* (RPR), *Pinelliae Rhizoma Praeparatum* (PRP), and *Pinelliae Rhizoma Praeparatum cum Zingibere et Alumine* (PRPZA) according to the different processing methods for toxicity reduction and pharmacological effect improvement. PRP is the product of RPR processed with alkaline solution and licorice. PRPZA is the product of RPR processed with alum and ginger juice. These excipients applied during processing can effectively eliminate or reduce irritant toxicity by decreasing the amount of needle-like raphides in RPR, which are popularly considered to be a major irritant component [7,8,9].

Although a large number of trials of PR have been carried out, the exact mechanism of its toxicity, especially its irritant effects, remains unclear. PR consists of multiple components, most of which are not easy to analyze. Modern phytochemical studies showed that the content of starch in PR is as high as 75% [10]. In addition, alkaloids, essential oils, amino acids, organic acids, and proteins are the chemical components of PR [7,11,12,13]. Unfortunately, the specific purified compounds derived from the herb were still not obtained. Previously, we had determined the abundant alkaloid trigonelline in coffee beans [14]. Trigonelline, distributed widely in plants, is also a major alkaloid in PR, but is not the toxic component. Other studies reported about PR’s toxic components and obtained different results, which have still not been recognized by authorities [10,15]. The raphides in RPR are currently regarded as a major irritant component; they are composed of calcium oxalate, protein, and trace amounts of polysaccharides [16]. Given the wide application of PR in Chinese clinical practice and unavailable studies about its exact toxicity mechanism, further research must be conducted to explore the underlying mechanism and provide useful information for a better understanding of PR.

Combination therapy is advocated based on the clinical settings and properties of each herb. For toxic herbs, co-therapy can help counteract their toxicities or side effects and simultaneously enhance their therapeutic effects [17,18]. PR, a representative toxic herbal medicine, can cause irritant toxicity when used as its raw form. Thus, RPR is commonly applied in its preparation forms, such as PRP or PRPZA, and frequently co-administered concomitantly with other chemical drugs because patients with cough, vomiting, or stagnation of phlegm require multiple drug therapy. Currently, PR and many chemical drugs are co-used to treat these diseases; these drugs include oxycodone [19], codeine [20], dextromethorphan [21], domperidone [22], chlorpromazine [23], and ambroxol [24], *etc.*, which are primarily or partially metabolized by cytochrome P450 isoenzyme 3A (CYP3A). Unfortunately, no study has reported the influence of PR on CYP3A. The safety assessment related to drug–drug interactions (DDIs) between PR and CYP3A-metabolizing drugs is essential and urgent.

In recent years, numerous DDIs between herbal medicines and chemical drugs have been documented [25,26,27]. The activity and expression of cytochrome P450 enzymes are closely related to DDIs. DDIs usually occur because of the inhibition or induction of CYPs. For example, the area under the plasma concentration–time curve and blood concentrations of cyclosporine, midazolam, tacrolimus, amitriptyline, digoxin, indinavir, warfarin, phenprocoumon, and theophylline can be significantly reduced after co-administration of St. John’s wort (*Hypericum perforatum*) via CYP3A induction [28]. CYP inhibition results in an undesirable elevation in plasma concentrations of co-administered drugs, causing adverse effects and toxicologically unsafe consequences [29]. CYP3A is the most abundant P450 enzyme in the liver, which is involved in the metabolism of 40%–50% of all currently used drugs [30]. However, CYP3A activity is frequently affected by its own substrates, and is recognized as one of the key factors causing DDIs [31]. In clinics, PR is frequently co-used with other herbal medicine or chemical drugs for treating diseases. Thus, the potential risk of DDIs between PR and other co-used drugs must be evaluated to provide important guidance for the clinical practice of PR.

**Figure 1:**
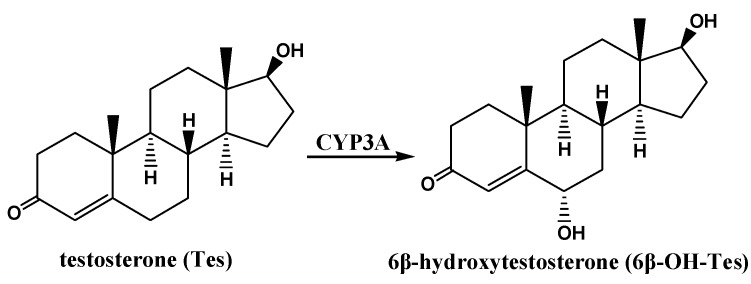
The major metabolic pathway of testosterone [32].

**Figure 2:**
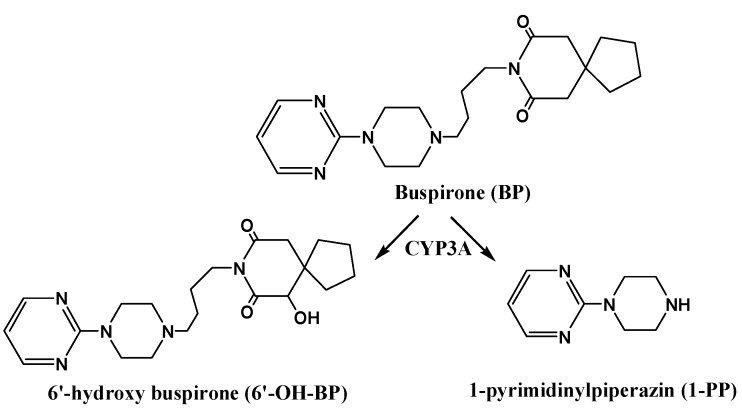
The major metabolic pathways of buspirone [35].

In the present study, CYP3A activity *ex vivo* and *in vivo* was examined after rats were pretreated with RPR, PRP, or PRPZA for 7 d using testosterone (Tes) and buspirone (BP), respectively, which have been accepted as specific probe substrates for CYP3A activity by the U.S. Food and Drug Administration (USFDA). Tes is primarily metabolized by CYP3A into 6β-hydroxytestosterone (6β-OH-Tes) (Figure 1) [32]. CYP3A activity in rat liver microsomes (RLMs) was determined by measuring the formation rate of 6β-OH-Tes using a validated ultra-performance liquid chromatography (UPLC) method [33,34]. BP is mainly metabolized by CYP3A into 1-(2-pyrimidinyl) piperazine (1-PP) and 6'-hydroxybuspirone (6'-OH-BP) (Figure 2) [35]. The pharmacokinetic characteristics of BP after pretreatment with PR were investigated. The concentrations of BP, 1-PP, and 6'-OH-BP in rat plasma were determined via a validated UPLC–tandem mass spectrometry method. Furthermore, western blot and real-time PCR were applied to explore the underlying mechanism of the changes in CYP3A activity.

## 2. Results and Discussion

### 2.1. Effects of RPR, PRP or PRPZA on Hepatic CYP3A Activity ex Vivo

#### 2.1.1. Effects of RPR on Hepatic CYP3A Activity *ex Vivo*

**Figure 3:**
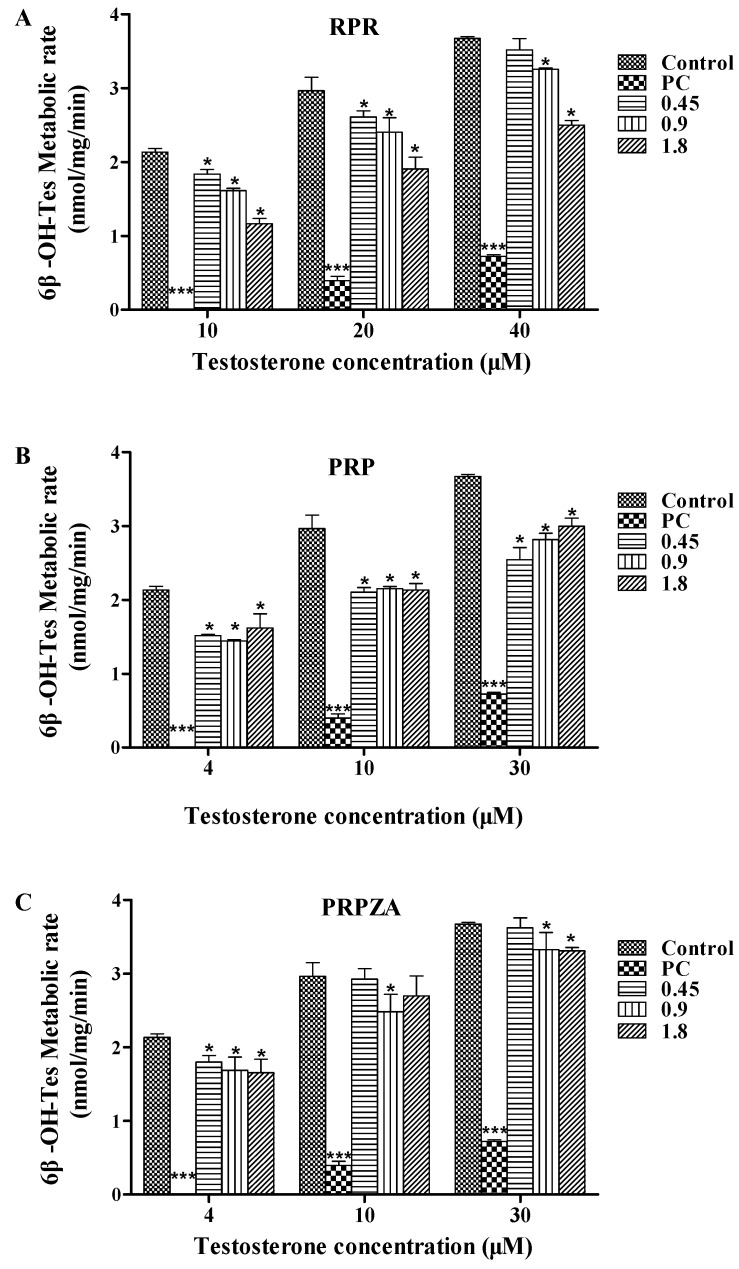
Formation rates of 6β-hydroxytestosterone (6β-OH-Tes) from testosterone (Tes) in rat liver microsomes (RLMs). Before the RLMs were prepared, rats were orally pretreated with RPR (**A**); PRP (**B**); PRPZA (**C**) (g·kg^−1^·day^−1^), or saline once daily for 7 days. The rats were orally pretreated with SKF-525A HCl (0.1 g·kg^−1^, 7 days) as the positive control (PC) for CYP3A inhibition. * *p* < 0.05 and *** *p* < 0.001 compared with the control group. Data are expressed as mean ± SD (*n* = 3).

Figure 3A shows the hepatic CYP3A activity observed in RLMs after 7 days of pretreatment with RPR (0.45, 0.9 or 1.8 g·kg^−1^·day^−1^, p.o.) or saline. Compared with that in the control group, the formation rates of 6β-OH-Tes from 4, 10, and 30 μmol·L^−1^ Tes in the 0.45 g·kg^−1^·day^−1^ dose group decreased by 14% (*p* < 0.05), 12% (*p* < 0.05), and 4% (*p* > 0.05), respectively; those in the 0.9 g·kg^−1^·day^−1^ dose group decreased by 24% (*p* < 0.05), 19% (*p* < 0.05), and 11% (*p* < 0.05), respectively; those in the 1.8 g·kg^−1^·day^−1^ group decreased by 45% (*p* < 0.05), 36% (*p* < 0.05), and 32% (*p* < 0.05), respectively.

#### 2.1.2. Effects of PRP on Hepatic CYP3A Activity *ex Vivo*

Figure 3B shows the hepatic CYP3A activity observed in RLMs after 7 days of pretreatment with PRP (0.45, 0.9 or 1.8 g·kg^−1^·day^−1^, p.o.) or saline. Compared with that in the control group, the formation rates of 6β-OH-Tes from 4, 10, and 30 μmol·L^−1^ Tes in the 0.45 g·kg^−1^·day^−1^ dose group decreased by 29% (*p* < 0.05), 29% (*p* < 0.05), and 31% (*p* < 0.05), respectively; those in the 0.9 g·kg^−1^·day^−1^ dose group decreased by 32% (*p* < 0.05), 27% (*p* < 0.05), and 23% (*p* < 0.05), respectively; those in the 1.8 g·kg^−1^·day^−1^ dose group decreased by 24% (*p* < 0.05), 28% (*p* < 0.05), and 18% (*p* < 0.05), respectively.

#### 2.1.3. Effects of PRPZA on Hepatic CYP3A Activity *ex Vivo*

Figure 3C shows the hepatic CYP3A activity observed in RLMs after 7 days of pretreatment with PRPZA (0.45, 0.9 or 1.8 g·kg^−1^·day^−1^, p.o.) or saline. Compared with that in the control group, the formation rates of 6β-OH-Tes from 4, 10, and 40 μmol·L^−1^ Tes in the 0.45 g·kg^−1^·day^−1^ dose group decreased by 16% (*p* < 0.05), 1% (*p* > 0.05), and 1% (*p* > 0.05), respectively; those in the 0.9 g·kg^−1^·day^−1^ dose group decreased by 21% (*p* < 0.05), 16% (*p* < 0.05), and 9% (*p* < 0.05), respectively; those in the 1.8 g·kg^−1^·day^−1^ dose group decreased by 23% (*p* < 0.05), 9% (*p* > 0.05), and 10% (*p* < 0.05), respectively.

As shown in Figure 3, the formation ratio of 6β-OH-Tes from Tes significantly decreased in the treated groups compared with that in the control group, suggesting that RPR, PRP, and PRPZA could inhibit CYP3A activity. Based on the results, we also found that different but regular inhibitory effects caused by PR were related to its processed forms. RPR at 1.8 g·kg^−1^·day^−1^ had the strongest inhibitory effects on CYP3A activity *ex vivo*. The processed products, especially PRPZA, only slightly inhibited CYP3A activity. Furthermore, some treated groups showed dosage dependency of the inhibitory effects. The inhibitory effects on CYP3A activity increased gradually with increasing dose of RPR. The PRP-treated groups showed an opposite tendency at 30 μmol·L^−1^ Tes that the inhibitory effects decreased gradually with rising dose. The PRPZA groups did not show marked dose-dependent inhibition on CYP3A activity. Therefore, the different processing methods of PR and different administration doses produced various but regular inhibitory effects on CYP3A. These findings may provide useful guidance for the clinical application of PR.

### 2.2. Effects of RPR, PRP or PRPZA on Hepatic CYP3A Protein and mRNA Expression Levels

#### 2.2.1. Effects of RPR, PRP or PRPZA on Hepatic CYP3A Protein Expression Levels

**Figure 4:**
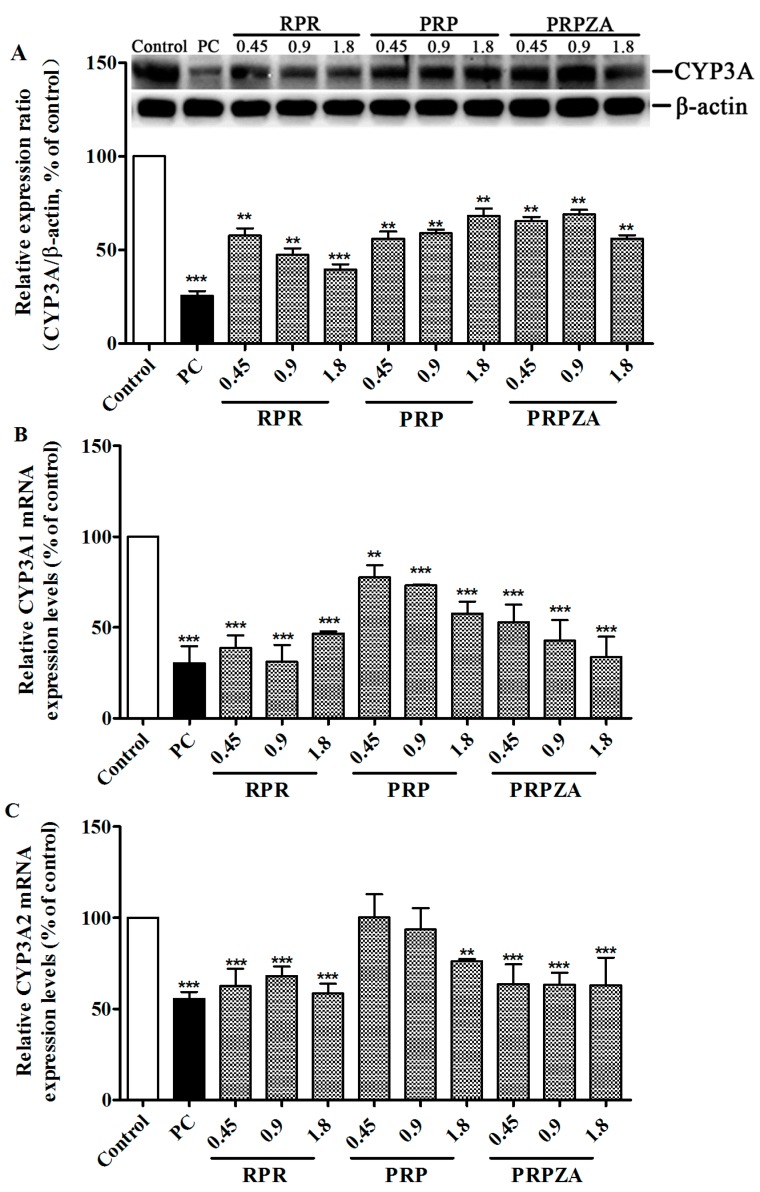
CYP3A protein (**A**); CYP3A1 mRNA (**B**) and CYP3A2 mRNA (**C**) expression levels in rat livers. Before the rat livers were prepared, rats were orally pretreated with RPR (A); PRP (B); PRPZA (C) (g·kg^−1^·day^−1^), or saline once daily for 7 days. The rats were orally pretreated with SKF-525A HCl (0.1 g·kg^−1^, 7 days) as the positive control (PC) for CYP3A inhibition. ** *p* < 0.01 and *** *p* < 0.001 compared with the control group. Data are expressed as mean ± SD (*n* = 3).

Figure 4A shows hepatic CYP3A protein expression levels in RLMs after 7 days of oral pretreatment with RPR/PRP/PRPZA (0.45, 0.9 or 1.8 g·kg^−1^·day^−1^), or saline. The ratio of CYP3A to β-actin concentration was used as the relative protein expression level. Compared with the control group, the CYP3A protein expression levels in the RPR group at 0.45, 0.9 and 1.8 g·kg^−1^·day^−1^ doses decreased by 42% (*p* < 0.01), 52% (*p* < 0.01) and 60% (*p* < 0.001), respectively; those in the PRP group decreased by 43% (*p* < 0.01), 41% (*p* < 0.01) and 31% (*p* < 0.01), respectively; those in the PRPZA group decreased by 34% (*p* < 0.01), 31% (*p* < 0.01) and 44% (*p* < 0.01), respectively.

#### 2.2.2. Effects of RPR, PRP or PRPZA on Hepatic CYP3A1 mRNA Expression Levels

Figure 4B shows hepatic CYP3A1 mRNA expression levels in rat livers after 7 days of the same pretreatment. CYP3A1 mRNA expression levels were determined by measuring the ratio of CYP3A1 to GAPDH. Compared with the control group, the CYP3A1 mRNA expression levels in the RPR group at 0.45, 0.9 and 1.8 g·kg^−1^·day^−1^ doses decreased by 61% (*p* < 0.001), 69% (*p* < 0.001) and 53% (*p* < 0.001), respectively; those in the PRP group decreased by 22% (*p* < 0.01), 27% (*p* < 0.001) and 42% (*p* < 0.001), respectively; those in the PRPZA group decreased by 47% (*p* < 0.001), 57% (*p* < 0.001) and 66% (*p* < 0.001), respectively.

#### 2.2.3. Effects of RPR, PRP or PRPZA on Hepatic CYP3A2 mRNA Expression Levels

Figure 4C shows hepatic CYP3A2 mRNA expression levels in rat livers after 7 days of the same pretreatment. CYP3A2 mRNA expression levels were determined by measuring the ratio of CYP3A2 to GAPDH. Compared with the control group, the CYP3A2 mRNA expression levels in the RPR group at 0.45, 0.9 and 1.8 g·kg^−1^·day^−1^ doses decreased by 37% (*p* < 0.001), 32% (*p* < 0.001) and 42% (*p* < 0.001), respectively; those in the PRP group decreased by −0.2% (*p* > 0.05), 6% (*p* > 0.05) and 23% (*p* < 0.01), respectively; those in the PRPZA group decreased by 36% (*p* < 0.001), 37% (*p < 0.001*) and 37% (*p* < 0.001), respectively.

CYP3A protein and mRNA levels were determined to explore the underlying mechanism of changes in CYP3A activity. The CYP3A isoforms in rat include CYP3A1, CYP3A2, and CYP3A23. CYP3A1 and CYP3A2 are the most versatile isoforms in rat liver, which is involved in the metabolism of currently used drugs. Thus, in this research, we focused on CYP3A1/2 in rats. However, given the high homology of CYP3A1 and CYP3A2 [36], we investigated the CYP3A protein and CYP3A1/2 mRNA expression levels. The results show that RPR, PRP, and PRPZA significantly down-regulated the CYP3A protein and CYP3A1/2 mRNA expression levels. We confirmed that CYP3A activity decreased because of the down-regulated expression levels of CYP3A protein and mRNA.

### 2.3. Effects of RPR, PRP or PRPZA on BP Pharmacokinetics in Vivo

**Figure 5:**
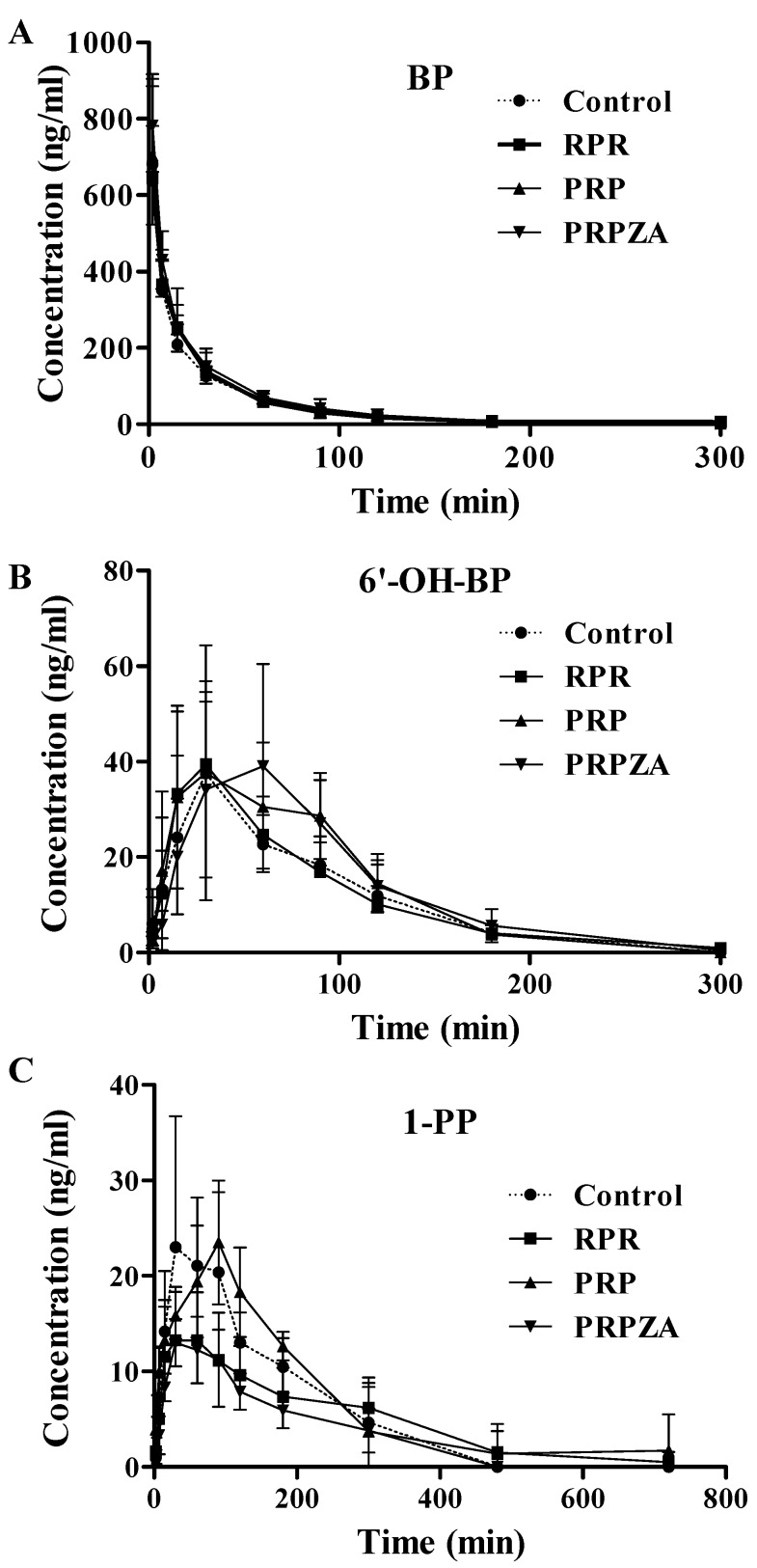
Concentration–time profiles of buspirone (BP) (**A**), 6'-hydroxybuspirone (6'-OH-BP) (**B**), and 1-(2-pyrimidinyl) piperazine (1-PP) (**C**) in rats after intravenous injection of 0.5 mg·kg^−1^ BP·HCl. Prior to intravenous injection of BP·HCl, the rats were orally pretreated with RPR*,* PRP, PRPZA, or saline once daily for 7 days. The data presented in this figure indicate mean ± SD (*n* = 5).

Based on the results *ex vivo*, we chose the medium dose (0.9 g·kg^−1^·day^−1^) as the treated dose to further study the influence of PR on CYP3A activity via BP metabolism *in vivo*. Figure 5 shows the mean plasma concentration-time profiles of BP, 1-PP, and 6'-OH-BP after oral pretreatment with RPR, PRP, PRPZA, or saline for 7 days before intravenous injection of 0.5 g·kg^−1^ BP·HCl. As shown in Table 1, the formation ratio of 6'-OH-BP from BP (AUC_0-t_ of 6'-OH-BP /AUC_0-t_ of BP) in the control group was 0.24 ± 0.06 [37], whereas those in the RPR, PRP and PRPZA groups were 0.24 ± 0.08, 0.28 ± 0.11 and 0.25 ± 0.05, respectively. The formation ratio of 1-PP from BP (AUC_0-t_ of 1-PP/AUC_0-t_ of BP) in the control group was 0.30 ± 0.11 [37], whereas those in the RPR, PRP and PRPZA groups were 0.22 ± 0.04, 0.31 ± 0.08 and 0.15 ± 0.03, respectively. Compared with that in the control group, the formation ratio of 1-PP from BP significantly decreased by 50% (*p* < 0.01) in the PRPZA group; other formation ratios of 6'-OH-BP or 1-PP from BP showed no evident change (*p* > 0.05).

**Table 1:** Pharmacokinetic parameters of buspirone (BP), 1-(2-pyrimidinyl) piperazine (1-PP) and 6'-hydroxybuspirone (6'-OH-BP) in rats after intravenous injection of 0.5 mg·kg^−1^ BP·HCl. Prior to intravenous injection of BP·HCl, the rats were orally pretreated with RPR, PRP, PRPZA (0.9 g·kg^−1^·day^−1^), or saline once daily for 7 days. The data presented in this table indicate mean ± SD (*n* = 5).

Variable	Control	RPR	PRP	PRPZA
**BP**				
*AUC*_0-t_ (μg·min·mL^−1^)	14,774.65 ± 2767.74	16,253.05 ± 4045.20	16,146.91 ± 953.18	17,502.24 ± 4206.67
*C*_max_ (ng·mL^−1^)	681.16 ± 253.80	672.76 ± 113.10	702.01 ± 181.79	783.03 ± 121.97
*t*_1/2_ (min)	118.15 ± 9.83	150.54 ± 34.79	146.12 ± 15.33	96.79 ± 8.02
*CL* (mL·min^−1^·kg^−1^)	34.37 ± 6.05	32.01 ± 7.99	30.53 ± 1.90	29.74 ± 7.03
*V*_d_ (L·kg^−1^)	5806.61 ± 752.26	7263.06 ± 3444.26	6436.20 ± 729.96	4175.27 ± 1125.25
**6'-OH-BP**				
*AUC*_0-t_ (μg·min·mL^−1^)	3515.02 ± 1032.97	3741.64 ± 811.23	4405.57 ± 1757.76	4468.04 ± 1787.44
*C*_max_ (ng·mL^−1^)	38.96 ± 17.86	40.05 ± 16.09	43.66 ± 22.07	41.43 ± 18.16
*t*_1/2_ (min)	43.62 ± 11.61	62.62 ± 21.72	38.23 ± 9.75	40.78 ± 10.74
*t*_max_ (min)	42.00 ± 26.83	27.00 ± 6.71	51.00 ± 36.12	60.00 ± 21.21
*CL* (mL·min^−1^·kg^−1^)	151.43 ± 39.84	139.88 ± 36.57	131.59 ± 58.71	126.79 ± 49.62
*V*_d_ (L·kg^−1^)	9667.04 ± 4169.74	13,323.56 ± 7985.70	7388.06 ± 3679.70	7013.16 ± 1855.36
**6'-OH-BP/BP ratio**	0.24 ± 0.06	0.24 ± 0.08	0.28 ± 0.11	0.25 ± 0.05
**1-PP**				
*AUC*_0-t_ (μg·min·mL^−1^)	4249.31 ± 1290.50	3624.86 ± 1142.96	4958.20 ± 1259.90	2596.72 ± 557.34
C_max_ (ng·mL^−1^)	27.35 ± 12.57	17.35 ± 7.20	25.99 ± 4.86	13.93 ± 3.87
*t*_1/2_ (min)	160.16 ± 52.85	244.73 ± 76.46	280.52 ± 308.26	268.82 ± 55.17
*t*_max_ (min)	48.00 ± 26.83	51.00 ± 29.24	78.00 ± 16.43	54.00 ± 25.10
*CL* (mL·min^−1^·kg^−1^)	126.20 ± 36.57	143.18 ± 56.52	97.07 ± 39.69	198.43 ± 34.45
*V*_d_ (L·kg^−1^)	30,074.20 ± 16,188.54	46,942.34 ± 11,037.16	25,588.78 ± 11,512.25	69,192.39 ± 18,332.26
**1-PP/BP ratio**	0.30 ± 0.11	0.22 ± 0.04	0.31 ± 0.08	0.15 ± 0.03 **

6'-OH-BP/BP ratio means AUC_0-t_ of 6'-OH-BP/AUC_0-t_ of BP; 1-PP/BP ratio means AUC_0-t_ of 1-PP/AUC_0-t_ of BP. ** *p* < 0.01 compared with control group.

The BP pharmacokinetic behaviors showed that the formation ratio of 1-PP from BP significantly decreased in the PRPZA group, but other formation ratios of 6'-OH-BP or 1-PP from BP did not markedly change. This finding demonstrates that PRPZA could inhibit CYP3A activity, but RPR and PRP had no significant effects on CYP3A activity *in vivo*. Conclusively, the inhibitory effects of RPR and PRP *ex vivo* were more evident than those *in vivo*. We presume that the inconsistent effects may be mainly attributed to the complex environment of rat *in vivo*, which will be explored urgently in the next research.

## 3. Experimental Section

### 3.1. Chemicals and Reagents

Buspirone HCl, testosterone (purity > 98%), 6β-hydroxytestosterone (purity > 98%), and Gemfibrozil (used as an internal standard, purity > 98%) were purchased from Sigma–Aldrich (St. Louis, MO, USA). 1-(2-pyrimidinyl) piperazine, 6'-hydroxybuspirone and SKF-525A HCl (used as a positive control drug) were obtained from Toronto Research Chemicals, Inc. (Toronto, ON, Canada). An NADPH regenerating system was purchased from BD Gentest Corp. (Woburn, MA, USA). Acetonitrile, dichloromethane, and formic acid were high-performance liquid chromatography grade. All other chemicals used were of analytical reagent grade or better.

### 3.2. Plant Material

*P. ternate* (Thunb.) Breit. (RPR, PRP, and PRPZA) were purchased from Huamiao Traditional Chinese Medicine Engineering Technology Development Center (Beijing, China) and confirmed by Institute of Chinese Materia Medica, China Academy of Chinese Medical Sciences.

### 3.3. Animals

Male Sprague–Dawley rats (180–220 g) were obtained by the Laboratory Animal Center of Guangzhou University of Chinese Medicine (Guangzhou, China; License: SCXK (yue) 2008-0020), and housed four per cage in a unidirectional airflow room under control (temperature: 23–25 °C; relative humidity: 40%–70%; 12 h light/dark cycle) with free access to standard rat food and water. All animal treatments followed guidelines for the care and use of laboratory animals and were approved by the ethics committee of Southern Medicine University (Guangzhou, China).

### 3.4. Preparation of Herbs

RPR, PRP or PRPZA (90 g) was immersed in 1800 mL of water for 30 min and then extracted with boiling water for 30 min. The residue was again extracted with 900 mL of boiling water for 30 min. The supernatant was combined and condensed to 500 mL. The prepared water extracts (10 µL) were diluted 100 times with methanol. The mixture was subsequently vortexed and then centrifuged at 18,000 *g* for 30 min, and the supernatant was subjected to Ultra High Pressure Liquid Chromatography-mass spectrometry-Quadrupole-time of flight (UHPLC-MS-Q-TOF, Agilent Technologies, Santa Clara, CA, USA) for analysis. The detailed information of analysis conditions, the chromatograms and possible ingredients in RPR, PRP and PRPZA water extracts were shown in the supplementary materials.

### 3.5. Preparation of Rat Liver Microsomes (RLMs)

Rats were randomly assigned to eleven groups with ten animals in each group. After oral pretreatment with RPR, PRP or PRPZA water extracts (1.8, 0.9 and 0.45 g·kg^−1^·day^−1^ as the high dose, medium dose and low dose, respectively), saline, or SKF-525A HCl (0.1 g·kg^−1^) for 7 day, the rats were anaesthetized with ethyl carbamate (50% w/v, 3 mL·kg^−1^, i.p.) on the eighth day, and the liver was excised. The livers of ten rats per group were combined to prepare RLMs. The detailed RLMs preparation procedures could be found in previous literature [38]. Bio-Rad protein assay (Bio-Rad, Hercules, CA, USA) was used to determine the concentration of microsomal protein with bovine serum albumin as the protein standard.

### 3.6. Determination of CYP3A Activity in RLMs by Tes Metabolism ex Vivo

The detailed information about determination of CYP3A activity by Tes metabolism was described in our previous study [37]. In brief, an NADPH regenerating system, including Tes (4, 10, and 30 μmol·L^−1^) and RLMs protein (0.02–0.05 mg·mL^−1^), was created to evaluate the formation rate of metabolite 6β-OH-Tes from Tes in RLMs with 7 d RPR, PRP or PRPZA pretreatment. The samples of Tes and 6β-OH-Tes were analyzed by a validated UPLC method described in our published paper [39]. CYP3A activity *ex vivo* was expressed as nanomoles of 6β-OH-Tes obtained per milligram of protein per minute. All experiments were run in triplicate.

### 3.7. CYP3A Protein Expression of RLMs by Western Blot

Eighty micrograms of total protein was separated by SDS-PAGE (4% stacking gel, 10% separating gel) and transferred from the gel to the PVDF membrane. Other detailed procedures were described in previous literature [37]. ECL chemiluminescence detection agent was applied to obtain the blot signals following the manufacturer’s instructions. The relative intensity of the protein bands was scanned and quantified using Quantity One Program (Bio-Rad, Hercules, CA, USA).

### 3.8. CYP3A1/2 mRNA Measurement by Real-Time PCR

After pretreatment, rat hepatic total RNA was isolated using the TRIzol extraction method (Invitrogen, Carlsbad, CA, USA). cDNA was synthesized from total RNA using a reverse transcription kit (TaKaRa, Shiga, Japan). SYBR Green real-time PCR amplification and detection were then performed using an ABI 7500 system (Applied Biosystems, Foster City, CA, USA). The forward and reverse primers of CYP3A1, CYP3A2 and GAPDH were displayed in previous research [37], which also described the PCR mixture and thermal profile. Target relative mRNA levels were normalized against GAPDH mRNA levels. All samples were run in triplicate.

### 3.9. Pharmacokinetics of BP in Rats with RPR, PRP or PRPZA Water Extracts Pretreatment for 7 days

Rats were randomly assigned to five groups with five animals in each group. After pretreatment with RPR, PRP or PRPZA water extracts (0.9 g·kg^−1^·day^−1^, p.o.) or saline for 7 days, the rats were given BP·HCl (0.5 mg·kg^−1^, single dose, i.v.). Serial blood samples were collected at 0, 2, 7, 15, 30, 60, 90, 120, 180, 300, 480, and 720 min after BP·HCl administration. The plasma samples were processed and analyzed by a validated UPLC/MS/MS method as described in our previous studies [35,37]. CYP3A activity *in vivo* was determined by measuring the formation ratios of 6'-OH-BP and 1-PP from BP (6'-OH-BP/BP and 1-PP/BP ratio values).

### 3.10. Data Analysis

Pharmacokinetic parameters were calculated using the standard non-compartmental method by Practical Pharmacokinetic Program Version 97 (3P97). All results were presented as mean ± standard deviation. Significant differences were analyzed using Student’s *t*-test (two groups) or one-way ANOVA followed by LSD test (for more than two groups) by SPSS 19.0. Statistical differences were considered significant at *p* < 0.05.

## 4. Conclusions

This study is the first to investigate the influence of RPR, as well as its processed products PRP and PRPZA, on CYP3A *ex vivo* and *in vivo*, which assessed the safety of co-administration of PR with other CYP3A-metabolizing drugs from the perspective of CYPs. Raw product RPR, as well as its processed products PRP and PRPZA can significantly inhibit CYP3A activity *ex vivo*. Such inhibition is due to the down-regulation of CYP3A protein and mRNA expression levels. Besides, the inhibitory effects caused by RPR and PRP *ex vivo* were more evident than that *in vivo*. Results of this study revealed that drug interaction between PR and the CYP3A-metabolizing drugs might be probable, suggesting that careful monitoring is essential for the concomitant use of PR with other drugs so as to avoid the adverse interactions. DDIs caused by PR-related co-therapy may be another factor that results in its toxicity. Considering no study has reported the index component in PR, so water extracts of PR were analyzed in this study based on clinical practices in China. Further research should be continued on the effects of bioactive ingredients in PR on CYP3A.

## Supplementary Materials

Supplementary materials can be accessed at: http://www.mdpi.com/1420-3049/20/01/0792/s1.
