# Supplementary material for: Pinelliae Rhizoma, a Toxic Chinese Herb, Can Significantly Inhibit CYP3A Activity in Rats
Source: Molecules. 2015 Jan 7;20(1):792–806. doi: 10.3390/molecules20010792 (PMC6272293; doi:10.3390/molecules20010792)
Supplement: Supplementary file 1 [file molecules-20-00792-s001.pdf]

Supplementary Materials

Identification of the Possible Ingredients in RPR, PRP and PRPZA Water Extracts by UHPLC-MS-Q-TOF

The prepared water extracts (10 μL) were diluted 100 times with methanol. The mixture was subsequently vortexed and then centrifuged at 18,000 *g* for 30 min, and the supernatant was subjected to UHPLC-MS-Q-TOF for analysis.

Chromatographic analysis was performed on an Agilent 1290 Infinity UHPLC system (Angilent, Santa Clara, CA, USA) equipped with a binary solvent delivery system, an auto-sampler and a MS-Q-TOF detector. Chromatographic separation was carried out on an Agilent ZORBAX Eclipse plus C18 column (1.7 μm 100 × 2.1 mm) at 30°C. The mobile phase consisted of (A) 0.1% acetic acid in water, and (B) acetonitrile. The eluting conditions were optimized as follows: isocratic at 10% B (0–3 min), linear gradient from 5% to 40%n B (3–10 min), 40% to 90% B (10–25 min), 90% to 90% B (25–28 min) and 90% to 10% B (28–30 min). The flow rate was set at 0.3 mL/min, the auto-sampler was maintained at 10 °C, and the sample injection volume was 5 μL.

The mass spectra were acquired using a 6540 Q-TOF mass spectrometer (Angilent Technologies, Santa Clara, CA, USA) equipped with an ESI interface in the positive ion mode (ESI+). The optimized mass spectrometric parameters were as follows: capillary voltage 4000 V, nebulizer gas pressure 50 psig, drying gas flow rate 11 L/min, gas temperature 350 °C, fragmentor and skimmer voltages 130 V and 50 V. The mass scale was calibrated using the calibration solution provided by the manufacturer between *m/z* 50 and 1500. A second orthogonal sprayer with a reference solution was used for continuous calibration using the following reference masses: *m/z* 121.0509 and 922.0098 in the positive ion mode. Masses were determined at the center of the mass peak using the width of the peak at half-peak height measured by computer-generated algorithms thus avoiding human bias.

A good chromatographic separation of constituents in RPR, PRP and PRPZA were achieved on a reversed-phase column using gradient elution with 0.1% formic acid and acetonitrile (Figure S1). And the information of possible ingredients in RPR, PRP and PRPZA were shown in Table S1.

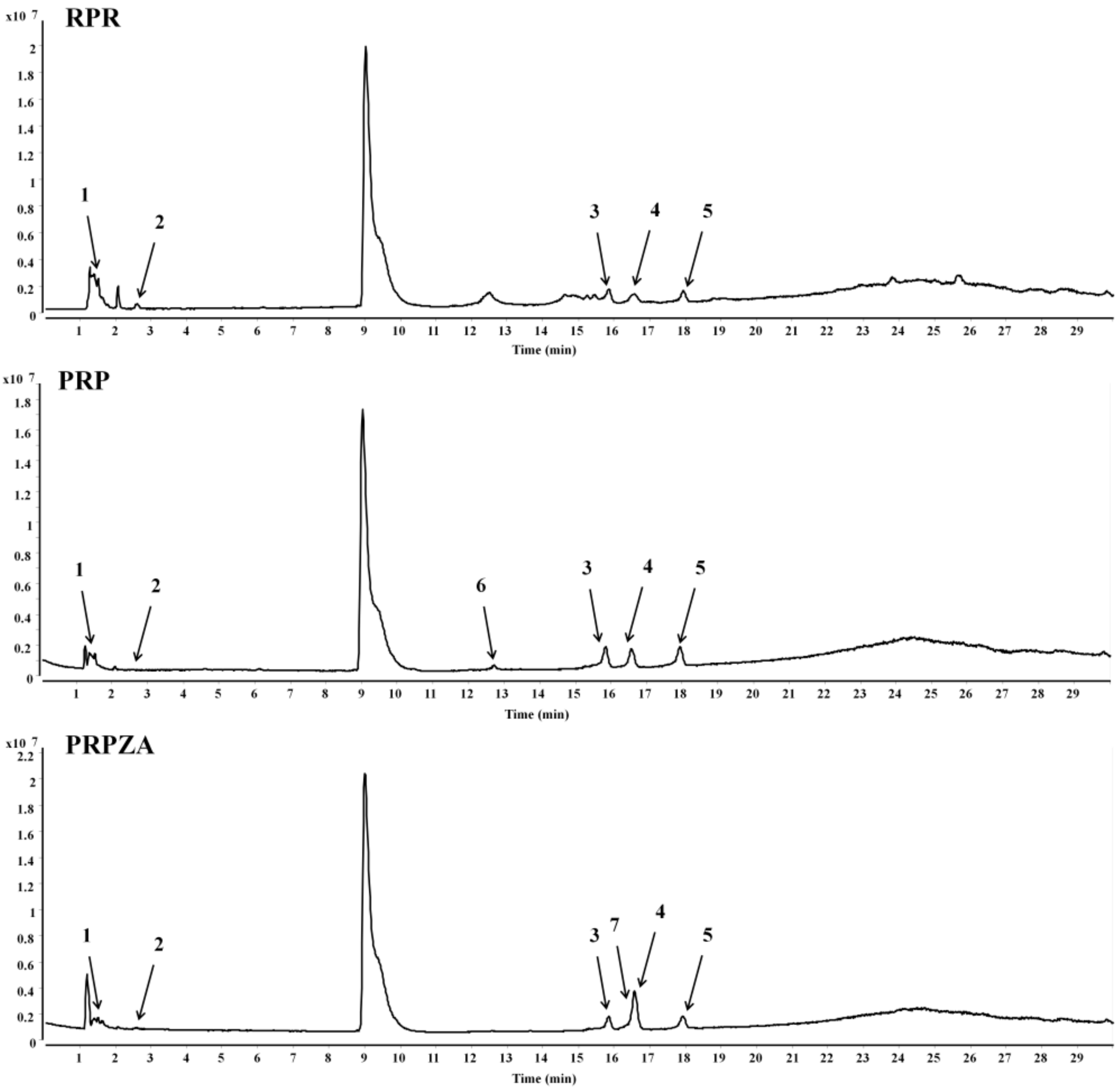

Figure S1. The chromatographic profile of RPR, PRP and PRPZA water extracts.

Table S1. Retention time (RT), HRMS data and molecular formula in RPR, PRP and PRPZA determined by UHPLC-MS-Q-TOF.

No.	RT (min)	*m/z* experimental	Formula	*m/z* calculated	Error (ppm)	Proposed Compound
1	1.44	138.0590	$C_7H_{15}NO_2$	138.0550	−0.57	Trigonelline
2	2.6	166.0862	$C_{10}H_{15}NO$	166.1226	3.34	Ephedrine
3	15.86	387.1806	$C_{22}H_{26}O_6$	387.1802	−0.87	Pinoresinol dimethyl ether
4	16.58	149.0256	$C_8H_4O_3$	149.0233	−0.98	Phthalic anhydride
5	17.91	437.1931	$C_{24}H_{30}O_6$	437.1935	1.02	Magnoshinin
6	12.72	823.4166	$C_{42}H_{62}O_{16}$	823.4111	−1.91	Glycyrrhizic acid
7	16.66	177.0553	$C_{17}H_{18}O_5$	177.0546	−0.36	Herniarin
